# System analysis based on the pyroptosis-related genes identifes GSDMD as a novel therapy target for skin cutaneous melanoma

**DOI:** 10.1186/s12967-023-04513-9

**Published:** 2023-11-10

**Authors:** Shixin Zhao, Yongkang Zhu, Hengdeng Liu, Xuefeng He, Julin Xie

**Affiliations:** 1 Department of Burns, The First Affiliated Hospital of Sun Yat-Sen University, Guangzhou, Guangdong China; 2 Institute of Precision Medicine, The First Affiliated Hospital, Sun Yat-Sen University, Guangzhou, Guangdong China

**Keywords:** Skin cutaneous melanoma, Pyroptosis, Prognostic model, Drug, GSDMD, Immune infiltration

## Abstract

**Background:**

Skin cutaneous melanoma (SKCM) is the most aggressive skin cancer, accounting for more than 75% mortality rate of skin-related cancers. As a newly identified programmed cell death, pyroptosis has been found to be closely associated with tumor progression. Nevertheless, the prognostic significance of pyroptosis in SKCM remains elusive.

**Methods:**

A total of 469 SKCM samples and 812 normal samples were obtained from The Cancer Genome Atlas (TCGA) and Genotype-Tissue Expression (GTEx) databases. Firstly, differentially expressed pyroptosis-related genes (PRGs) between normal samples and SKCM samples were identified. Secondly, we established a prognostic model based on univariate Cox and LASSO Cox regression analyses, which was validated in the test cohort from GSE65904. Thirdly, a nomogram was used to predict the survival probability of SKCM patients. The R package “pRRophetic” was utilized to identify the drug sensitivity between the low- and high-risk groups. Tumor immune infiltration was evaluated using “immuneeconv” R package. Finally, the function of GSDMD and SB525334 was explored in A375 and A2058 cells.

**Results:**

Based on univariate Cox and LASSO regression analyses, we established a prognostic model with identified eight PRGs (AIM2, CASP3, GSDMA, GSDMC, GSDMD, IL18, NLRP3, and NOD2), which was validated in the test cohort. SKCM patients were divided into low- and high-risk groups based on the median of risk score. Kaplan–Meier survival analysis showed that high-risk patients had shorter overall survival than low-risk patients. Additionally, time-dependent ROC curves validated the accuracy of the risk model in predicting the prognosis of SKCM. More importantly, 4 small molecular compounds (SB525334, SR8278, Gemcitabine, AT13387) were identified, which might be potential drugs for patients in different risk groups. Finally, overexpression of GSDMD and SB525334 treatment inhibit the proliferation, migration, and invasion of SKCM cells.

**Conclusion:**

In this study, we constructed a prognostic model based on PRGs and identified GSDMD as a potential therapeutic target, which provide new insights into SKCM treatment.

**Supplementary Information:**

The online version contains supplementary material available at 10.1186/s12967-023-04513-9.

## Introduction

Skin cutaneous melanoma (SKCM) is the most aggressive skin cancer characterized by poor prognosis, resulting in 57,043 new deaths and 324,635 new cases of SKCM in 2020 worldwide [1, 2]. Although the 5-year relative survival rate of patients with localized SKCM by surgical resection is 98% [3]. However, once systemic metastasis occurs, the 5-years survival rate falls to 23% [4]. Furthermore, some advanced SKCM exhibits insensitivity to radiotherapy and chemotherapy due to its high aggressiveness [5]. Presently, clinical trials have demonstrated the efficacy of immunotherapy and targeted therapy, which have become the basic means of systemic therapy [6, 7]. In spite of the rapid development of these therapeutic approaches, there are limitations due to the heterogeneity of SKCM. Patients with identical stages and treatments may exhibit varying prognoses and treatment responses [8, 9]. Thus, it is imperative to identify prognostic biomarkers for decision-making.

Pyroptosis is a type of programmed cell death that is triggered by inflammatory caspases. This process is mainly dependent on the activation of the caspase family, which subsequently cleaves and activates Gasdermin proteins. Subsequently, the activated Gasdermin proteins translocate to the cell membrane, where it forms pores that cause cellular swelling, membrane rupture, and ultimately, the release of cytoplasmic contents, culminating in pyroptotic cell death [10]. Inflammasomes have been confirmed to be involved in diverse hallmarks of tumor development and progression, exerting either pro-tumorigenic or anti-tumorigenic effects [11]. Specifically, the cleavage of IL-1β and IL-18 precursors triggers the synthesis and release of other inflammatory factors, thereby amplifying the local and systemic inflammatory response through pyroptosis [12]. The expression of ASC protein in metastatic melanoma exhibits a significant reduction as compared to primary melanoma [13]. The NLRP3 inflammasome has been shown to promote drug resistance of oral squamous cell carcinoma (OSCC) to 5-fluorouracil [14]. Therefore, targeting NLRP3 may represent a promising strategy for 5-fluorouracil adjuvant chemotherapy in this cancer. In addition, it was found that knockdown of AIM2 inhibits cell growth and promotes apoptosis in OSCC [15].

As an inflammatory and programmed form of cell death, pyroptosis can not only impede tumor cell proliferation, but also create a tumor microenvironment (TME) conducive to cell growth [16]. Hence, there is no universal conclusion regarding the relationship between pyroptosis and tumors. Therefore, it is imperative to conduct a comprehensive investigation into the role of pyroptosis in SKCM tumorigenesis and progression, and establish a relevant prognostic model of pyroptosis, to facilitate the treatment of SKCM. In the present study, we aimed to construct a prognostic model utilizing pyroptosis-related genes (PRGs) to predict the prognosis of SKCM patients. Moreover, we conducted experiments to validate the function of GSDMD in A375 and A2058 cell lines, which could potentially serve as a therapeutic target for SKCM treatment. Our study systematically investigated the prognostic significance of PRGs and their associations with clinical characteristics, providing insight into SKCM treatment.

## Materials and methods

### Data retrieval

The RNA sequencing (RNA-seq) data of 469 SKCM samples and 812 normal tissues, along with their respective clinicopathological parameters, were obtained from the cancer genome atlas (TCGA, https://portal.gdc.cancer.gov) and Genotype-Tissue Expression (GTEx, https://www.gtexportal.org/home/) databases. Additionally, the RNA-seq data and clinicopathological features of 214 SKCM samples were acquired from Gene Expression Omnibus GSE65904 datasets (https://www.ncbi.nlm.nih.gov/). Prior to subsequent analysis, gene expression data were normalized using the “Sanger box” tools (http://sangerbox.com/).

### Identification of PRGs

By referring to the pyroptosis-related literature, we obtained 33 candidate PRGs for subsequent analysis after removing the duplicates (Additional file 1: Table. S1) [16–19].

### Identification of differentially expressed PRGs

The differentially expressed PRGs in 469 SKCM samples and 812 normal samples were identified using the “DESeq2” R package, with a cut-off value of *P* < 0.05 and |log2 fold change (FC)| > 1.5. The volcano of PRGs and heatmap of differentially expressed PRGs was visualized through the use of the “ggplot” R package. Additionally, protein–protein interactions (PPI) were drawn using the String database (https://string-db.org/) and Cytoscape software (Cytoscape, 3.7.2), with an interaction score of > 0.4. Finally, cBioPortal (https://www.cbioportal.org/) was used to identify the PRGs alteration frequency and mutation type.

### Functional enrichment analysis of differentially expressed PRGs

The 23 differentially expressed PRGs were then subjected to functional enrichment analysis. The functions of Gene Ontology (GO), including biological process (BP), cell composition (CC), and molecular function (MF), as well as Kyoto Encyclopedia of Genes and Genomes (KEGG), were analyzed and visualized using the “clusterProfiler” and “ggplot2” R packages.

### Identification of prognostic PRGs

The training set utilized in this study comprised of 469 SKCM samples and 812 normal samples obtained from the TCGA and GTEx databases. To explore the correlation between the PRGs and the overall survival (OS) of SKCM patients, we performed a univariate Cox regression analysis using the “survival” R package, with the threshold set at *P* < 0.05 for further analysis. To eliminate gene collinearity and reduce the number of genes, we employed LASSO Cox regression. Finally, we conducted a multivariate Cox regression analysis based on the results of the univariate Cox regression.

### Construction and validation of prognostic model based on PRGs

The standardized SKCM mRNA expression data in the train set was utilized to calculate the risk score. The detailed formula was described as follows.

$$Riskscore\, = \,\sum\limits_{i}^{n} {xiyi}$$

The coefficient of PRGs in LASSO Cox regression analysis was represented by X, while Y represented the expression levels of corresponding PRGs. Subsequently, SKCM patients were categorized into low- and high-risk groups based on the median risk score, and the analysis of OS between these two groups was performed. The prognostic efficiency of the model was evaluated by time-dependent Receiver Operating Characteristic (ROC) curves using the “timeROC” R package. To make the model more convincing, we utilized GSE65904 datasets as the test cohort for validation. The expression of each PRGs was also normalized, and subsequently, the risk score was calculated using the above formula. Patients in the test cohort were also stratified into low- and high-risk groups based on the median risk score, and comparison of their OS was conducted. Next, time-dependent ROC curves were also constructed to evaluate the prognostic efficiency of the prognostic model.

### Construction of nomogram and calibration curves

In order to predict individual survival probability, “RMS” R package was utilized to construct the nomogram, and subsequently, calibration curves for the prediction of 1-, 5-, and 10-year survival rate of SKCM patients were plotted.

### Drug sensitivity analysis

The sensitivity score of each small molecular compounds for patients in both high-risk and low-risk groups was calculated using the “pRRophetic” R package. Subsequently, the 3D conformations of the compounds were visualized through the utilization of the PubChem website (https://pubchem.ncbi.nlm.nih.gov/).

### Tumor microenvironment analysis

The infiltration of immune cells, and the expression of immune checkpoints (ICs) were analyzed by “immuneeconv” R package, which integrates six latest algorithms, including TIMER, xCell, MCP-counter, CIBERSORT, EPIC and quanTIseq.

### Prediction of PRGs prognostic model on the effect of immunotherapy

The immunophenoscore (IPS) is a widely used predictor of immunotherapeutic response by quantifying determinants of tumor immunogenicity [20]. This algorithm relies on the assessment of immune-related genes encompassing MHC-associated molecules, checkpoints or immunomodulators, effector cells, and suppressor cells. By quantifying these genes and assigning them equal weights, the IPS is constrained within a range of 0–10, with higher scores indicating greater immunogenicity.

### Tissue samples and ethics statement

To further verify the difference of protein expression levels between SKCM and normal skin, 10 pairs of SKCM tissues and normal skin samples were collected and subsequently subjected to paraffin embedding for immunohistology and immunofluorescence. This study followed the Helsinki Declaration and received approval from the Ethics Committee Board of the First Affiliated Hospital of Sun Yat-Sen University (IIT-2022-474). Written informed consent was obtained from all participants.

### Immunohistology (IHC) assay

The paraffin-embedded tissues mentioned above were sectioned, followed by deparaffinization and rehydration. Subsequently, EDTA was used for antigen repair. After that, the slides were incubated with GSDMD antibody solution (A18281, 1:200, ABclonal, China) at 4 ℃ overnight. On the second day, the slides were incubated with corresponding HRP-conjugated secondary antibodies (ab6721, 1:1000, Abcam, UK) for 1 h at 37 ℃. Image acquisition was done using a digital pathology section scanner (Kfbio, Ningbo, China).

### Immunofluorescence (IF) assay

Cell samples were fixed in 4% paraformaldehyde for 15 min at room temperature. Both the cell samples and tissue slides were then washed, permeabilized, and blocked. Thereafter, cell samples and tissue slides were incubated with primary antibodies and then with an Alexa Fluor 555-conjugated secondary antibody (ab150078, 1:500, Abcam, UK) or Alexa Fluor 488-conjugated secondary antibody (ab150077, 1:500, Abcam, UK). The nuclei were counterstained with DAPI (Beyotime Biotechnology, Shanghai, China). The following primary antibodies were used: anti-GSDMD (A18281, 1:200, ABclonal, China), anti-Ki67 (A11390, 1:200, ABclonal, China), anti-CD4 (ab133616, 1:200, Abcam, UK). For actin staining, ActinGreen 488 ReadyProbes (R37110, 1:20, Invitrogen, USA) was added to the fixed cells, and the cells were then incubated for 30 min at room temperature. Images were captured using a confocal microscope (Zeiss, Oberkochen, Germany).

### Cell culture

A375 and A2058 cell lines were purchased from iCell Bioscience Inc. (Shanghai, China). All cell lines were cultivated in Dulbecos Modified Eagle Medium (DMEM; Gibco, USA) supplemented with 10% fetal bovine serum (FBS; Gibco, USA) at 37 ℃ with 5% CO_2_.

### Lentiviral construction, infection and generation of stable cell lines

We constructed GSDMD-overexpressing lentivirus (OE-GSDMD) using the pLV-EF1A-hGSDMD plasmids (VectorBuilder Inc., Guangdong, China), and a scramble sequence was designed as a negative control (OE-Control). Subsequently, A375 and A2058 cell lines were infected with corresponding lentivirus using polybrene (GeneChem, Shanghai, China) according to the manufacturer’s instructions. After 72 h of infection, 2 μg/mL puromycin was used to select stable clonal cell lines.

### Cells treatment with SB525334

Cells were trypsinized and plated, then allowed to attach overnight. According to the previous study, we used 1 μmol/L SB525334 to act on SKCM cells for 12 h [21]. Then the medium was changed for the follow-up study.

### Western blot (WB) analysis

The collected cells was lysed with ice-cold RIPA lysis buffer supplemented with a protease inhibitor cocktail, and centrifuged for 10 min after sonication. After concentration detection, equal amounts of protein (20 μg) were separated by 10% SDS-PAGE and then electrotransferred onto polyvinylidene fluoride membranes, which were incubated overnight at 4 ℃ with anti-GSDMD (A18281, 1:500, ABclonal, China), and anti-GAPDH (5174S, 1:1000, CST, USA) after blocked HRP-conjugated secondary antibodies (ab6721, 1:10000, Abcam, UK) for 1 h at with 5% BSA. Thereafter, the membranes were incubated with corresponding room temperature after removing excess primary antibodies. The signal was detected using FluorChem E system (ProteinSimple, USA).

### Flowcytometry (FCM) assay

The cell death in each group of A375 and 2058 cell lines was measured by using the FCM assay based on PE Annexin V and 7-Amino-Actinomycin (7AAD) staining. Briefly, cells in each group were incubated with PE Annexin V/7AAD for 15 min at room temperature in the dark. Subsequently, the cells were gathered for FCM analysis, wherein a total of 10,000 events were examined. Three independent experiments were performed.

### CCK8 assay

Cell viability was evaluated by CCK8 assay. Briefly, OE-Control group, OE-GSDMD group and SB525334 group of A375 and A2058 cell lines in the logarithmic growth phase were trypsinized, resuspended in complete medium, and cultured overnight. According to the manufacturer's protocol, Cell Counting KIT-8 reagent (Abcam, UK) was used the next 4 days to assess cell viability. Finally, the optical density at 462 nm at each time point was detected by microplate reader.

### Ki67 staining

Ki67 staining was used to determine the proliferative capacity of cells. When the fusion rate reached about 70%, the cells were fixed with 4% paraformaldehyde for 15 min at room temperature, which was followed by washing, permeabilizing and blocking. Ki67 staining was conducted with anti-Ki67 antibody (1:200; ab15580, Abcam, USA). Images were captured using a confocal microscope (Zeiss, Oberkochen, Germany).

### Transwell assay

Cells were harvested, resuspended in serum-free medium, and then plated in the upper chamber of transwell (Corning, NY, USA) for migration assays or plated in the upper chamber coated with Matrigel (Corning, NY, USA) for invasion assays. Complete medium with 10% FBS was added into the lower chamber and incubated for 24 h at 37 ℃. Thereafter, cells were wiped from the top surface of the chamber. Cells on the bottom surface were fixed with 4% paraformaldehyde for 10 min and stained with crystal violet (Servicebio, Wuhan, China) for 5 min. The number of migrating or invading cells was imaged and counted.

### Statistical analysis

We used two-tailed Student's t-test for comparisons between two groups, and one-way ANOVA for comparisons between multiple groups. *P* < 0.05 was considered statistically significant. Three or more independent replicates were used for each experiment. The results were presented as mean ± standard deviation (SD).

## Results

### Identifcation of diferentially expressed PRGs in SKCM

**Fig. 1:**
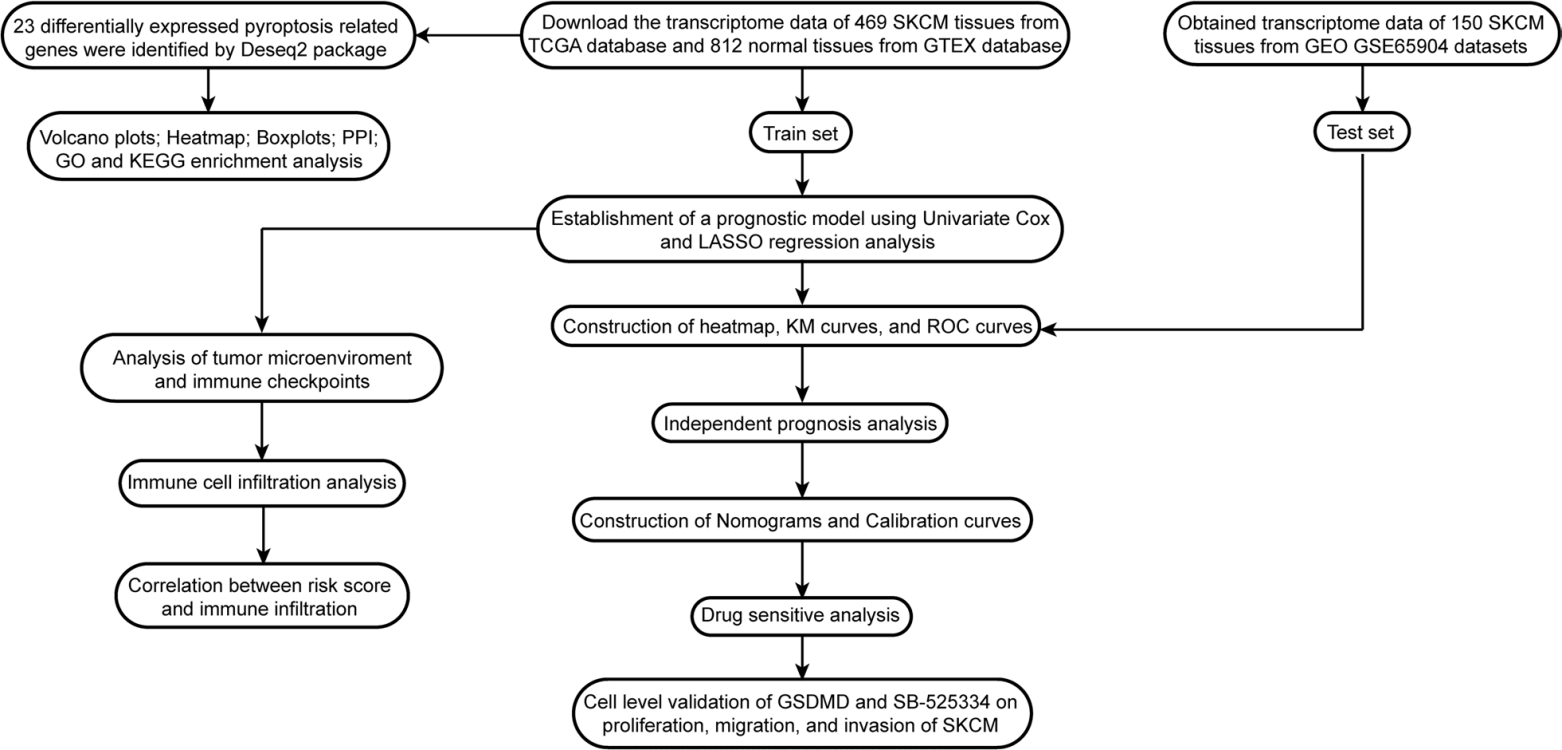
Flow chart of the analysis process

**Fig. 2:**
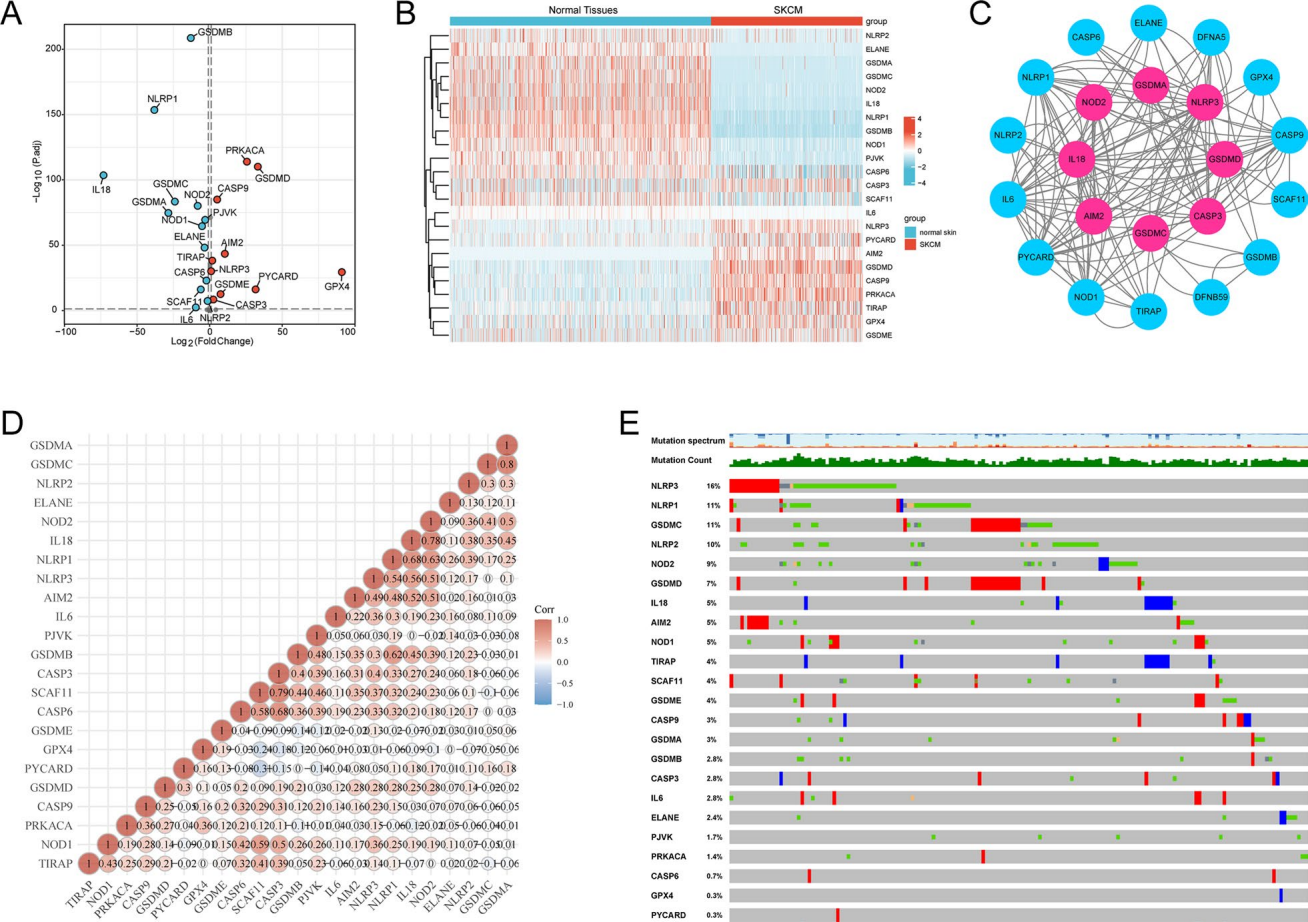
Differentially expressed pyroptosis-related genes (PRGs) between SKCM tissues and normal tissues. **A** Volcano plot shows PRGs, with red dots indicating high expression and blue dots indicating low expression in SKCM tissues. **B** Heatmap of diferentially expressed PRGs, with red denoting high expression, blue denoting low expression. **C** Protein–protein interaction (PPI) network indicates the interaction of PRGs (interaction score = 0.4). **D** Correlation matrix of interaction in PRGs, with red dots indicating positive correlation, and blue dots indicating negative correlation. **E** Summary of alterations in differentially expressed PRGs in SKCM.

The detailed workflow of this study was shown in (Fig. 1). We obtained 469 SKCM patients from TCGA database and 812 normal tissues from GTEx database. A total of 23 differentially expressed PRGs were identifed based on the cutoff criteria of |log_2_ FC | > 1.5 and *P* < 0.05 from 33 PRGs using R package “DESeq2”. Volcano plots and heatmaps showed that 10 PRGs were significantly up-regulated, whereas 13 PRGs were down-regulated in SKCM (Fig. 2A, B). The correlations among these 23 differentially expressed PRGs were shown in PPI network and correlation matrix (Fig. 2C, D). Furthermore, mutations in SKCM patients have been observed in these differentially expressed PRGs (Fig. 2E).

### Functional enrichment analysis

In order to understand the functions of differentially expressed PRGs, we conducted GO and KEGG pathway enrichment analyses. Based on GO enrichment analyses, these differentially expressed PRGs were mostly associated with pyroptosis, inflammasome complex, and cysteine-type endopeptidase activity involved in apoptotic process. Additionally, the analysis of KEGG indicated that these differentially expressed PRGs were associated with pertussis, legionellosis, and NOD-like receptor signaling pathway (Additional file 1: Fig. S1; Table. S2). These results suggested that differentially expressed PRGs were involved in other biological processes besides pyroptosis.

### Construction of a prognostic model based on PRGs in the training cohort

**Fig. 3:**
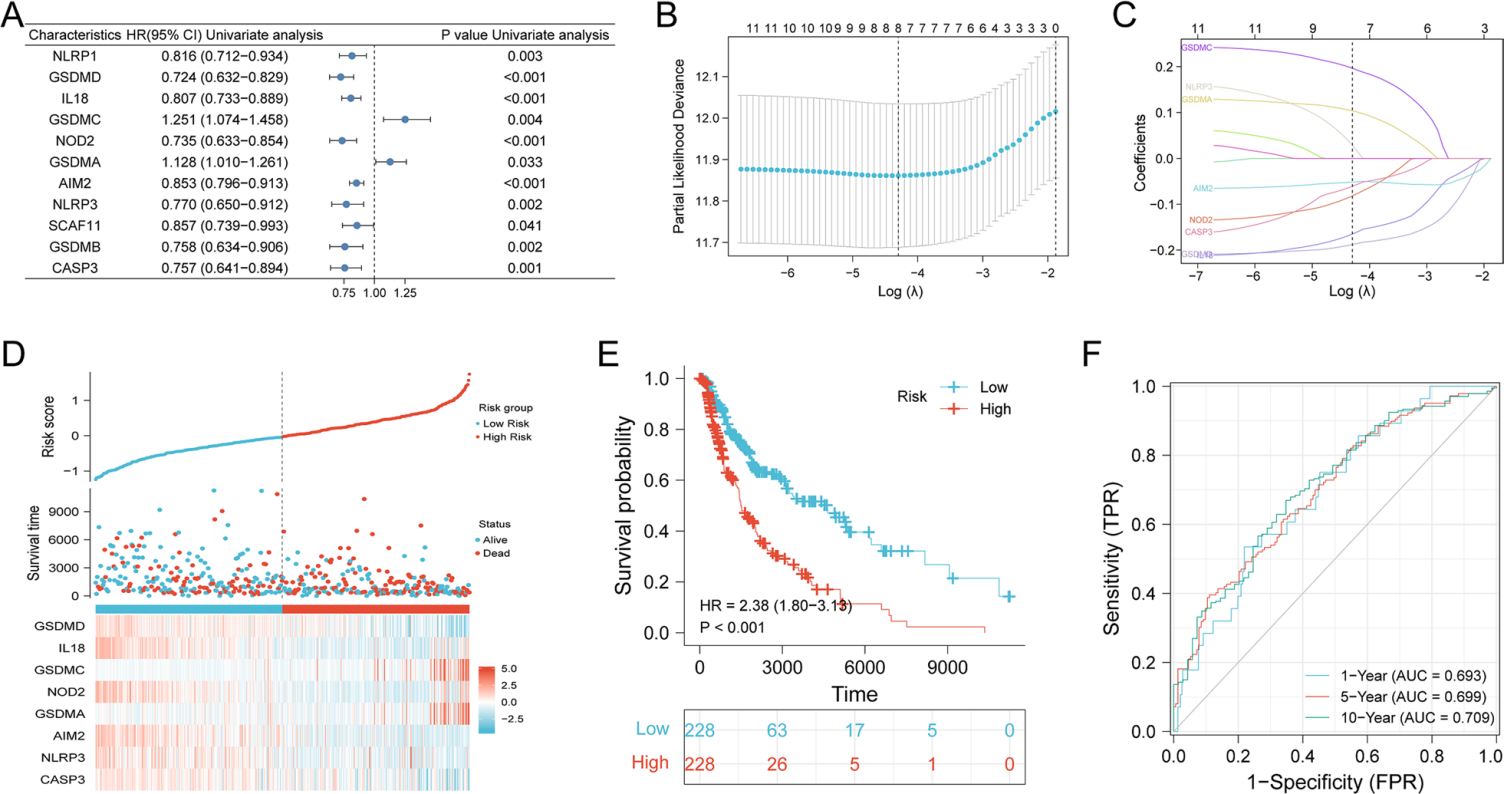
Construction of a risk prognostic model based on PRGs in the TCGA cohort. **A** Forest plots for hazard ratios (HRs) of differentially expressed PRGs in SKCM. **B** Partial likelihood deviance versus log (λ) was plotted using LASSO Cox regression model. **C** Coefficients of selected features are shown by lambda parameter. **D** Risk score analysis of 8 PRGs prognostic signature. In the top panel, patients were evenly divided into two groups based on the median risk score, with blue indicating the low-risk group, and red indicating the high-risk group. The middle panel shows the survival status of patients with SKCM, with blue dots representing survival, and red dots representing death. The bottom panel depicts the expression of the eight PRGs, with blue indicating the low-risk group, and red indicating the high-risk group. **E** Kaplan–Meier survival curves showing the overall survival of patients in the high-risk and low-risk groups. **F** Time dependent ROC curves verifying the predictive efficiency of the risk score

In order to construct a prognostic model based on PRGs, we first conducted univariate COX regression analysis. 13 patients were excluded due to missing survival outcome or survival time, leaving 456 patients for subsequent analysis. As shown in Fig. 3A, we identified 11 PRGs with *P* < 0.05, including two potential risky genes (GSDSA, GSDSC) and nine potential protective genes (NLRP1, GSDMD, IL18, NOD2, AIM2, NLRP3, SCAF11, GSDMB, CASP3). Based on the results of univariate Cox regression, we then performed LASSO regression analysis and identified eight PRGs as candidate prognostic factors, including GSDMA, GSDMC, GSDMD, NLRP3, IL18, NOD2, AIM2, and CASP3 (Fig. 3B, C). Thereafter, we constructed the PRGs prognostic model using these eight PRGs. Specifically, the PRGs-based prognostic model was formulated as follows: Risk score = [GSDMC × 0.1964] + [GSDMA × 0.1037] + [NLRP3 × 0.0283] + [GSDMD × (− 0.1879)] + [IL18 × (-0.1649)] + [NOD2 × (− 0.0816)] + [AIM2 × (− 0.0518)] + [CASP3 × (− 0.0608)].

In order to validate whether this PRGs prognostic model could predict the prognosis of SKCM patients, we divided the 456 patients into a low-risk group (*n* = 228) and a high-risk group (*n* = 228) according to the threshold of median risk score. In comparison with low-risk group, patients in high-risk group had a higher mortality rate and shorter survival time, indicating that higher risk scores were associated with worse prognosis. GSDMD, IL18, NOD2, AIM2, NLRP3, and CASP3 were lowly expressed in the high-risk group, while GSDMA, GSDMC were highly expressed in the high-risk group (Fig. 3D). Kaplan–Meier survival curves also demonstrated that patients in the high-risk group had a worse prognosis (Fig. 3E). Time dependent ROC curves indicated that the prognostic accuracy of OS was 0.693 at 1-year, 0.699 at 5-year, and 0.709 at 10-year (Fig. 3F). All the above results illustrated that the PRGs prognostic model had excellent accuracy for predicting prognosis in the training cohort.

### Validation of the prognostic model in the test cohort

**Fig. 4:**
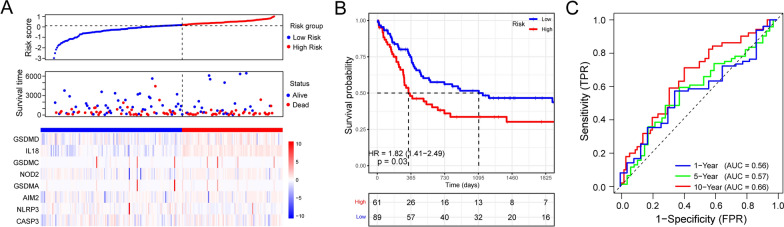
Validation of the risk prognostic model based on PRGs in the test cohort. **A** Risk score analysis of 8 PRGs prognostic signature. In the top panel, patients were evenly divided into two groups based on the median risk score, with blue indicating the low-risk group, and red indicating the high-risk group. The middle panel shows the survival status of patients with SKCM, with blue dots representing survival, and red dots representing death. The bottom panel depicts the expression of the eight PRGs, with blue indicating the low-risk group, and red indicating the high-risk group. **B** Kaplan–Meier survival curves showing the overall survival of patients in the high-risk and low-risk groups. **C** Time dependent ROC curves verifying the predictive efficiency of the risk score

In order to confirm the accuracy of the PRGs prognostic model, 150 SKCM patients from GSE65904 were obtained and calculated the risk score using the same formula in the training cohort. Based on the median risk score, 89 patients were classified as low risk, whereas 61 patients were classified as high risk in the test cohort. As expected, patients in the low-risk group had a lower mortality rate and longer OS time than those in the high-risk group, suggesting that lower risk scores were associated with better prognosis (Fig. 4A). Moreover, Kaplan–Meier survival analysis also illustrated that patients in different risk group had different prognosis, which was consistent with the result in training cohort (Fig. 4B). Finally, time dependent ROC curves demonstrated that the prognostic accuracy of OS was 0.560 at 1-year, 0.570 at 5-year, and 0.660 at 10-year (Fig. 4C). Altogether these findings indicated that the established prognostic model had acceptable accuracy of predicting prognosis in the test cohort.

### Construction of nomogram and calibration curves

**Fig. 5:**
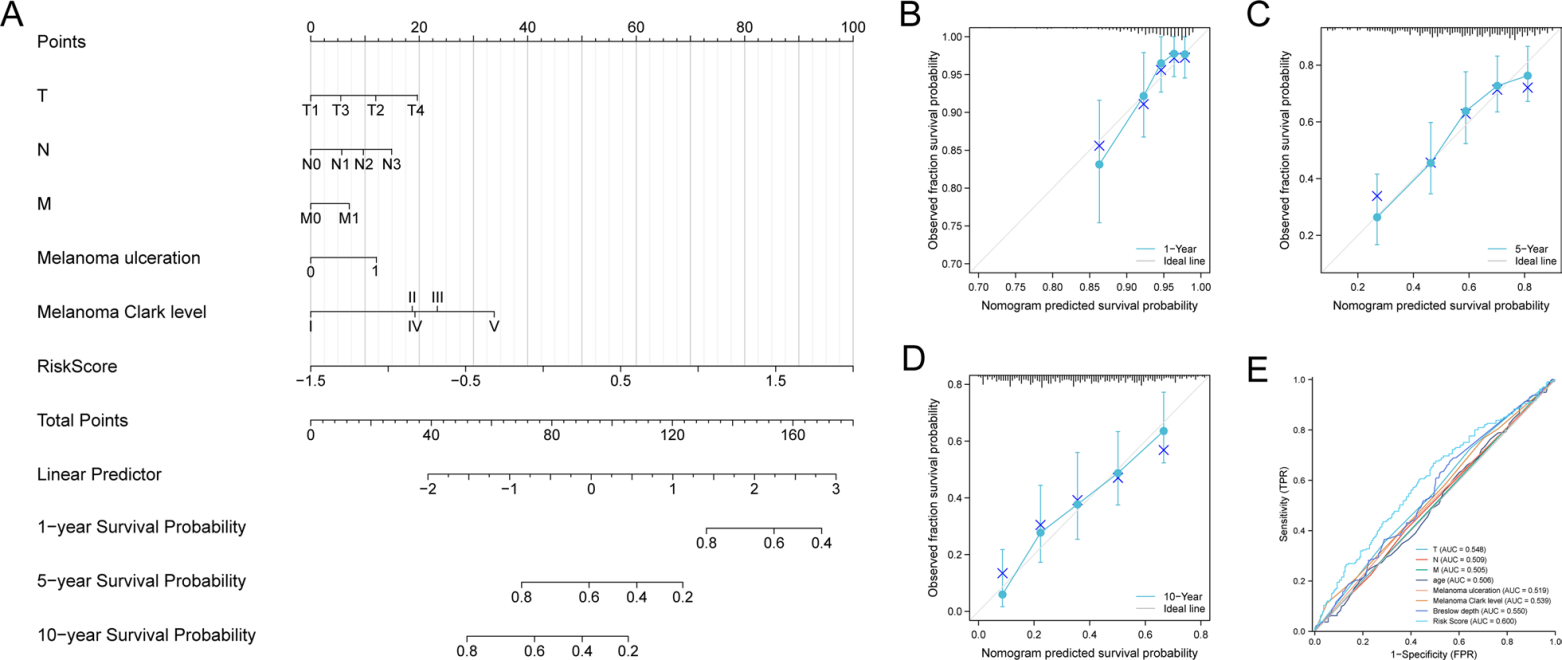
Nomogram to predict survival probability of patients with SKCM. **A** Nomogram combining risk score with pathologic characteristics. **B**–**D** Calibration plots for predicting 1-, 5-, 10-year overall survival of SKCM patients. **E** ROC curves for prediction of overall survival by the risk score and other variables (T stage, N stage, M stage, age, melanoma ulceration, melanoma Clark level, and Breslow depth)

In order to provide clinicians with a more accurate quantitative method for predicting the prognosis of SKCM patients, we developed a nomogram that integrates T stage, N stage, M stage, melanoma ulceration, melanoma Clark level and risk scores. Among the various clinical parameters, risk score emerged as the most significant factor in our established nomogram (Fig. 5A). Moreover, we constructed calibration curves, which proved the nomogram's superior prediction efficiency (Fig. 5B-D). Compared with traditional prognostic scoring systems, the model we established had the highest AUC value (AUC = 0.600, Fig. 5E). All the above results demonstrated that the nomogram incorporating our risk scores can be utilized to precisely forecast the prognosis of SKCM patients.

### Drug sensitivity analysis in the low- and high- risk group

**Fig. 6:**
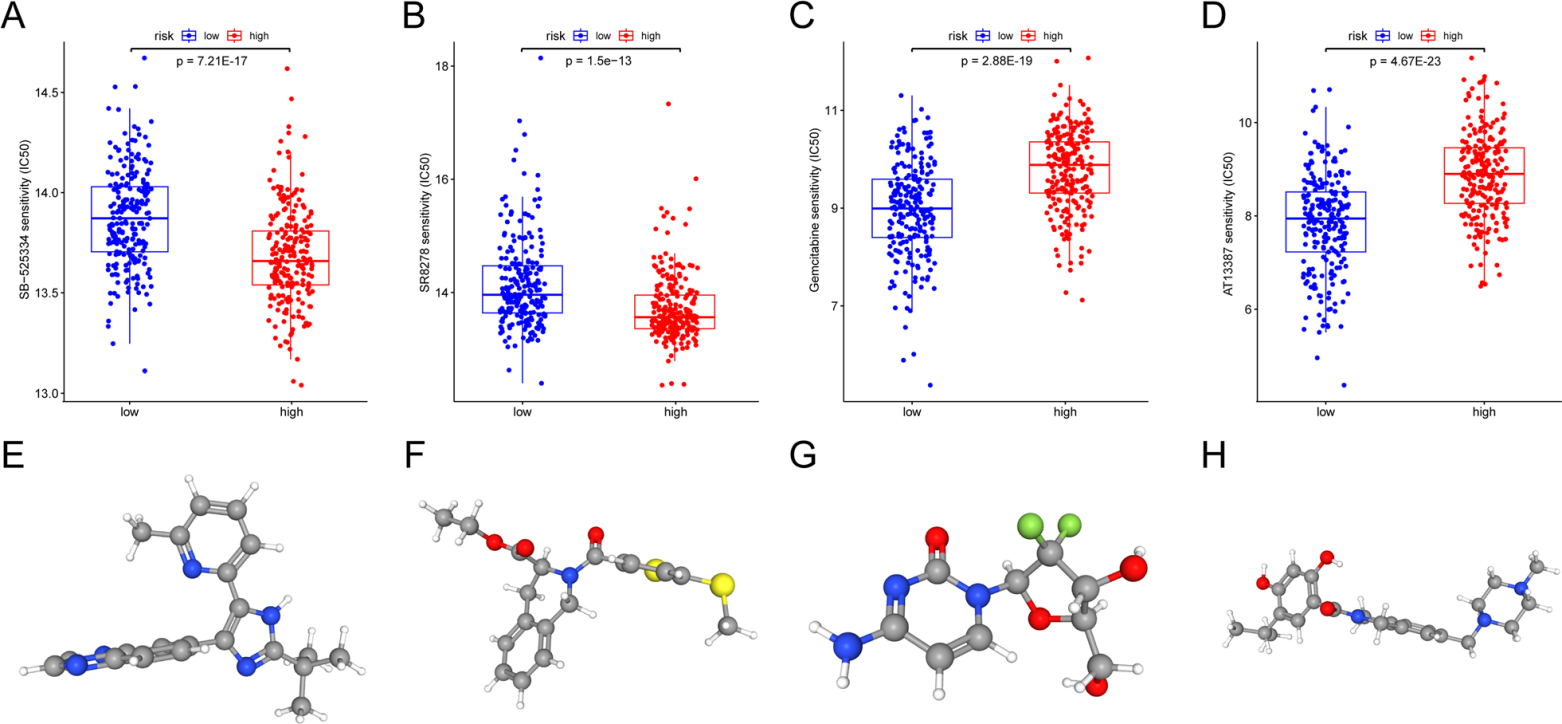
The screened drugs for SKCM treatment. IC50 value of SB525334 (**A**), SR8278 (**B**), Gemcitabine (**C**), AT13387 (**D**) in high- and low-risk patients with SKCM. The corresponding 3D structures are shown in **E**, **F**, **G** and **H**, respectively

In order to estimate chemotherapy response, the half-maximal inhibitory concentration (IC50) available in the GDSC database (https://www.cancerrxgene.org/) for SKCM patients was calculated by the “pRRophetic” R package. There were 47 small molecular compounds that exhibited significantly different responses between the low- and high-risk groups (Additional file 1: Table. S3). The top four small molecular compounds were found to have the most significant fold change between the low- and high-risk groups, including SB525334 (log FC = − 0.192, Fig. 6A), SR8278 (log FC = − 0.442, Fig. 6B), Gemcitabine (log FC = 0.815, Fig. 6C), AT13387 (log FC = 1.024, Fig. 6D). SKCM patients in the low-risk group exhibited greater sensitivity to Gemcitabine and AT13387, whereas those in the high-risk group displayed heightened responsiveness to SB525334 and SR8278. PubChem website (https://pubchem.ncbi.nlm.nih.gov/) was then used to visualize the 3D conformations of these four small molecules (Fig. 6E–H). Based on these findings, these small molecular compounds may be effective in treating SKCM. In summary, our findings provide promising molecular chemotherapy agents for individuals diagnosed with SKCM.

### Kaplan–Meier curves of prognostic genes

To investigate the correlation between the expression of eight PRGs included in the prognostic model and SKCM prognosis, we generated Kaplan–Meier curves for these genes. The findings showed that high expression levels of AIM2, GSDMD, IL18, NLRP3, and NOD2 were associated with favorable prognosis in SKCM patients, while the expression levels of CASP3, GSDMA, and GSDMC did not exert a significant impact on the prognosis (Additional file 1: Fig. S2).

### Protective PRGs screening and functional identification

**Fig. 7:**
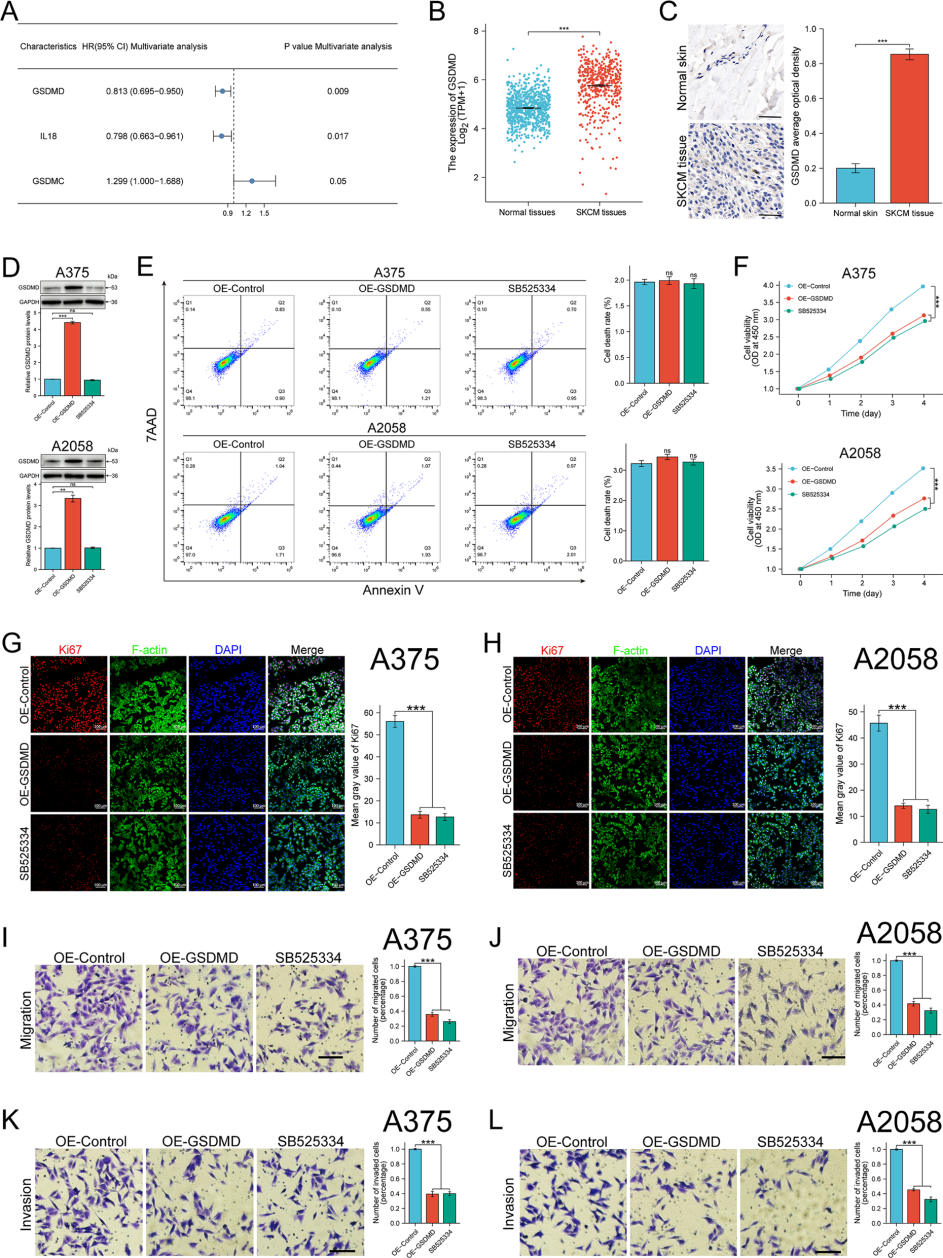
GSDMD is overexpressed in SKCM tissues as a protective gene by inhibiting the proliferation, migration and invasion abilities of SKCM cells. **A** Multivariate Cox regression analysis was performed on the genes derived from the univariate Cox regression analysis. **B** Comparison of mRNA levels of GSDMD in SKCM and normal tissues from TCGA and GTEx databases. **C** Representative images of GSDMD immunohistochemistry and its quantification. **D** WB and WB quantification of GSDMD protein levels in A375 and A2058 cell lines infected with scramble or GSDMD overexpression lentivirus, or treated with SB525334. **E** Cell apoptosis in each group of A375 and 2058 cell lines was measured by FCM assay. **F** Cell viability in each group of A375 and 2058 cell lines was determined by CCK8 assay. **G**–**H** Ki67 immunofluorescence staining in A375 (**G**) and A2058 (**H**) cell lines and its quantification (Scale bar = 100 µm). **I**–**J** The migration ability of A375 (**I**) and A2058 (**J**) cell lines was detected by Transwell assay (Scale bar = 100 µm). **K**–**L** The invasion ability of A375 (**K**) and A2058 (**L**) cell lines was detected using Matrigel-coated Transwell cell culture chambers (Scale bar = 100 µm). All results are presented as the mean ± SD, ns: *p* > 0.05; ***p < 0.001

In order to identify prognostic factors for SKCM, multivariate Cox regression analysis was conducted by using the significant prognostic factors identified in univariate Cox regression analysis. A total of three PRGs were identified, two of which were potential protective genes and one of which was potential risk gene (Fig. 7A). As a core pyroptosis gene, the mean level of GSDMD mRNA expression in SKCM was higher than that in normal skin (Fig. 7B). The augmented expression of GSDMD was also immunohistochemically confirmed (Fig. 7C). Moreover, there was a significantly better outcome in the group characterized by high GSDMD expression, while patients with tumors characterized by low GSDMD expression faired worse (Additional file 1: Fig. S2E).

To investigate the role of GSDMD in SKCM cells and evaluate the potential therapeutic effects of SB525334, we overexpressed GSDMD in A375 and A2058 cell lines or exposed these two cell lines to 1 μmol/L of SB525334 for 12 h. WB results showed that the GSDMD protein was significantly increased in GSDMD overexpression group (OE-GSDMD), while SB525334 treatment exerted little effect on the expression of GSDMD (Fig. 7D). Subsequently, cell death and cell viability were analysed by FCM and CCK8 assays. FCM analysis indicated that overexpression of GSDMD or SB525334 treatment did not trigger obvious cell death in SKCM cells (Fig. 7E). However, CCK8 assays showed that the viability of the two cell lines was decreased in OE-GSDMD group and SB525334 treatment group (Fig. 7F). Immunofluorescent staining of Ki67 also confirmed that the expression of Ki67 in SKCM cells was significantly reduced in OE-GSDMD and SB525334 group, indicating overexpression of GSDMD and SB525334 treatment decrease cell proliferation (Fig. 7G, H). Transwell migration assay was conducted to detect the migration ability. The results showed that GSDMD overexpression and SB525334 treatment reduced the migration ability of SKCM cells (Fig. 7I, J). Moreover, cell invasion ability was detected using Matrigel-coated Transwell chambers and the results also showed GSDMD overexpression and SB525334 treatment reduced the invasive ability of SKCM cells (Fig. 7K, L). All the above results illustrated that GSDMD overexpression and SB525334 treatment could inhibit cell proliferation, migration and invasion in SKCM.

### Analysis of tumor microenvironment and immunotherapy

**Fig. 8:**
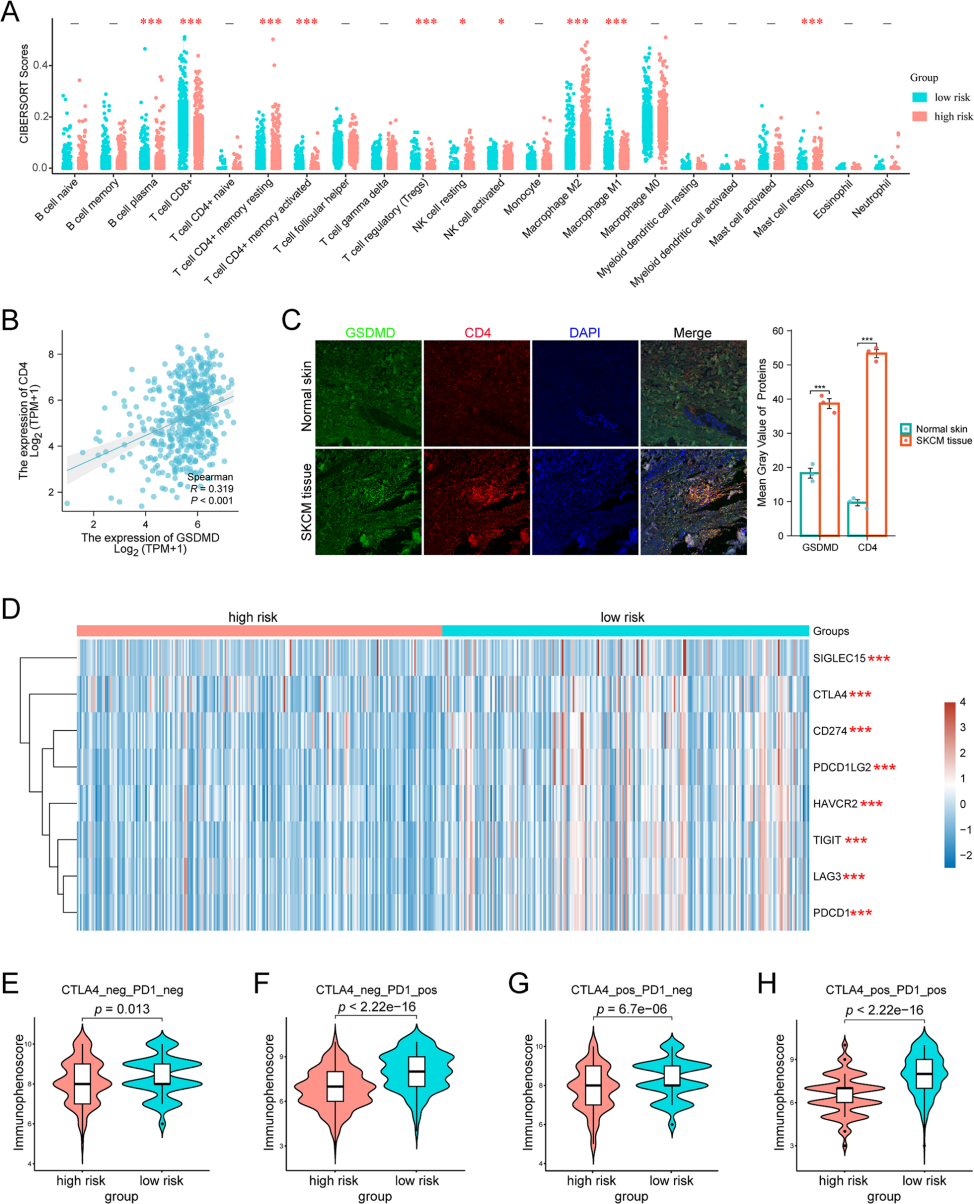
Analysis of tumor microenvironment and immunotherapy. **A** Dotplots of 22 immune cells, with blue dots indicating low-risk group and red dots indicating high-risk group. **B** Scatter plots showing the relationship between GSDMD and CD4. **C** Representative images of immunofluorescence and quantitative analysis demonstrated that both GSDMD and CD4 were overexpressed in SKCM tissues (*n* = 12). **D** Heatmap showing the expression of eight immune checkpoints in the low- and high-risk groups. **E**–**H** The IPS scores between low- and high-risk groups when CTLA-4 or/and PD1 positive. *p < 0.05,***p < 0.001

In recent years, immunotherapy has revolutionized cancer treatment, prolonging patients' survival time. Therefore, the association between pyroptosis-related risk score and immune cell infiltration in SKCM was evaluated. The degree of 22 immune cells infiltration in SKCM patients was calculated by CIBERSORT tool and a threshold of *P* < 0.05 was considered as cut-off criteria. The results showed that the infiltration levels of B cell plasma, T cells CD8, T cell CD4 memory activated, T cell regulatory, NK cell activated, and macrophage M1 in the high-risk group were lower than those in the low-risk group, whereas the infiltration levels of NK cell resting, T cell CD4 memory resting, mast cell resting, and macrophage M2 in the high-risk group were higher than those in the low-risk group (Fig. 8A). Thereafter, to investigate the reliability of the risk score-related immune cell infiltration, we further analyzed the relationship between GSDMD expression and CD4 expression. Spearman correlation analysis demonstrated a significant positive correlation between GSDMD levels and CD4 levels (Fig. 8B). Furthermore, our immunofluorescence analysis demonstrated that GSDMD was highly co-expressed with CD4 in SKCM tissues (Fig. 8C). Collectively, these findings indicated that pyroptosis has the potential to efficiently modulate the TME and elicit a robust T cell-mediated immune response against tumors.

In addition to immune cells, ICs are also considered as a crucial part in immunotherapy. In this study, the association between the risk score and ICs was analyzed. The results showed a notable disparity in the expression of ICs between the low- and high-risk groups, with almost all ICs exhibiting high expression levels in the low-risk group (Fig. 8D). These findings indicated that patients belonging to distinct risk groups may exhibit varying responses to immunotherapy. Currently, IPS is an extensively employed algorithm for immune response prediction. Therefore, we categorized all patients into 4 groups based on their PD1 and CTLA4 expression: CTLA4_negative_PD1_negative, CTLA4_negative_PD1_positive, CTLA4_positive_PD1_negative, and CTLA4_positive_PD1_positive, respectively. The results showed that all four groups of patients with low risk score had higher IPS scores (Fig. 8E–H). Consequently, patients classified in the low-risk group may exhibit more robust immunogenic phenotype, making them more appropriate candidates for ICs blockade therapy.

## Discussion

Pyroptosis, a caspase-dependent pro-inflammatory programmed cell death, are well documented to play an important role in tumorigenesis and tumor development [10, 22, 23]. Available evidence indicates that pyroptosis plays a distinct role in different tumors [24–26]. On the one hand, pyroptosis has the potential to facilitate the demise of tumor cells; on the other hand, the release of inflammatory cytokines during cell death may foster a conducive microenvironment that serves as a hotbed for tumor proliferation [27]. Specifically, overexpression of GSDMB in breast cancer has been found to be associated with tumor progression, which indicated unfavorable response to targeted treatment of HER-2 [28]. In addition, GSDMA was identified as a tumor suppressor gene in gastric cancer, whereas GSDMB was observed to be overexpressed and exhibited oncogenic properties [29, 30]. Furthermore, GSDMD was found to be up-regulated in non-small cell lung cancer, and its elevated expression was associated with tumor metastasis and poor prognosis [31]. Recently, Zhu et al. using the network analysis, found that pyroptosis in SKCM was closely related to tumor stemness, TME, ICs levels, response to ICs blockade, and prognosis, suggesting that pyroptosis plays a crucial role in SKCM [32]. The objective of this study was to establish a prognostic model of PRGs, facilitating the diagnosis and prognosis prediction of SKCM patients.

Based on univariate Cox and Lasso regression analysis, we constructed a prognostic risk model using 8 genes (AIM2, CASP3, IL18, NLRP3, NOD2, GSDMA, GSDMC, GSDMD). AIM2, the inflammasome sensor, can activate caspase-1 in an ASC-dependent manner [33]. Studies have demonstrated that decreased expression of AIM2 promotes hepatocarcinoma progression by activating mTOR-S6K1 pathway [34]. CASP3 was previously thought to execute apoptosis, but it is now believed to induce pyroptosis by cleaving GSDME to form N-GSDME [35]. CASP3 depletion suppresses GSDME-dependent pyroptosis in lung cancer cells [36]. Inflammasomes and cytokines are also believed to play crucial roles in oncogenesis, including proliferation, metastasis, and invasion [37]. It has been shown that IL-18 promotes the growth, angiogenesis, and metastasis of melanoma both in autocrine and paracrine manners [38]. NLRP3 senses a variety of stimulus, and promotes the maturation and secretion of IL-1β/ IL-18, eventually leading to pyroptosis [39, 40]. Studies have shown that NLRP3 deletion could significantly reduce the lung metastasis of melanoma by activating NK cells [41]. NOD2 was found to be dysregulated in melanoma and high expression of NOD2 predicts a better prognosis for melanoma patients, which is consistent with the KM survival curve [42]. Gasdermin superfamily, the executioner of pyroptosis, is composed of GSDMA/B/C/D/E and PJVK. GSDMA is expressed in the suprabasal epidermis and is associated with epidermal differentiation and cornification [43]. GSDMC is up-regulated and proved to be associated with poor prognosis both in breast cancer and lung adenocarcinoma [44, 45], which is consistent with the manifestations in our constructed model. GSDMD is also one of the most important model genes in our prognostic model. Canonical pyroptotic death is composed of GSDMD cleavage and IL-1β and IL-18 release [46]. Studies have demonstrated that GSDMD is dysregulated in gastric cancer and lung cancer, and is associated with tumor proliferation, metastasis, and immune microenvironment [31, 47, 48]. However, studies on GSDMD in SKCM remain scarce. Therefore, cell experiments were performed to identify the specific role of GSDMD in SKCM. Our results showed that overexpression of GSDMD significantly suppressed proliferation, migration, and invasion of SKCM cells. However, GSDMD, as a key effector of pyroptosis, did not induce prominent cell death, which may be related to the lack of upstream effectors cleaving GSDMD to the active N-GSDMD [49]. Therefore, we speculated that overexpression of GSDMD may be associated with better prognosis by inhibiting SKCM growth and metastasis. In recent years, great progress has been made in the study of recombinant proteins. Gao et al. used recombinant human hair keratin nanoparticles to accelerate dermal wound healing [50]. In the future, we may also be able to use recombinant proteins to increase intratumoral GSDMD content to improve patients prognosis.

In addition, we screened out four potential small molecular compounds targeted different patient cohorts, including SB525334, SR8278, Gemcitabine, and AT13387. SR8278, antagonist of circadian clock gene REV-ERBα, has been shown to possess the capacity to modulate the reaction of tumor cells to cisplatin chemotherapy [51]. Estrogen-induced pituitary adenoma could be inhibited by SR8278 through reducing the expression of PER2 [52]. It has been demonstrated that the FDA approved antitumor drug Gemcitabine plays a vital role in retardation of melanoma growth, enhancing CD8 + T-cell immune response, and thus boosting antitumor immunity [53]. As a small-molecule HSP90 inhibitor, AT13387 has long sustained antitumor activity in melanoma, and combination of AT13387 with BRAF/MEK inhibition could delay the emergence of drug resistance [54]. There is substantial evidence indicating that the hyperactivation of TGF-β/Smad signaling plays a significant role in promoting epithelial-mesenchymal transition and tumor metastasis in various malignancies, including SKCM [55]. Moreover, TGF-β has been found to directly suppress immune populations [56]. SB525334, a TGF-β receptor inhibitor, exhibits promising therapeutic potential in the treatment of breast cancer and pancreatic cancer by inducing neutrophil polarization towards an antitumor phenotype [57, 58]. Additionally, it has been demonstrated that SB525334 could inhibit the self-renewal, migration, and invasion of ovarian cancer stem cells by blocking TGF-β pathway [59]. The present study also showed that SB525334 suppressed the proliferation of SKCM cells and inhibited the migration and invasion ability. Regrettably, the non-specific accumulation in non-tumor organs retarded its application clinically. To date, no clinical trials of SB525334 in melanoma are in progress. However, the recent preclinical data suggested intravenous injection of cyto-pharmaceuticals loaded with SB525334 or intratumoral injection of degradable mesoporous silica nanoparticles loaded with SB525334 could specifically deliver drugs to sites of interest without non-specific distribution [57, 58]. Also as a solid tumor, patients with SKCM may benefit from similar drug delivery system.

The immunotherapeutic approach for SKCM has evolved from cytokine-based intervention to PD-1 immune checkpoints. This shift has resulted in substantial improvements in overall survival of patients with SKCM. Studies have demonstrated that the future treatment of SKCM should be combination of chemotherapy with immunotherapy [60]. Increasing studies have shown that CD4 + T cells is critical for productive anti-tumor responses through recognition of antigens in melanoma [61]. The infiltration of “T cell CD4 memory activated” in high-risk subgroup was lower than that in low-risk subgroup in our study, which is consistent with previous study [62]. Moreover, previous research has showed that M1 macrophages are associated with anti-tumor immunity, while M2 macrophages are associated with melanoma genesis and invasion [63]. The present study showed that a decrease in M1 macrophages was accompanied by an increase in M2 macrophages in patients in the high-risk group. Differences in immune cell infiltration probably explains why patients in the high-risk group have poor survival prognosis. In addition, the combination of anti-PD-1 and anti-CTLA-4 has been authorized as a first line therapy for individuals with unresectable or metastatic melanoma, which achieved an objective response rate of 59% [64, 65]. The present study illustrated that patients in the low-risk group had higher expression level of ICs and IPS scores, indicating that patients in the low-risk group had a stronger immunogenic phenotype and were more suitable for immune checkpoint blockade treatment.

Whilst these findings construct a prognostic model and identify GSDMD as a potential therapeutic target, it is prudent to consider the potential limitations. First, the molecular mechanism underlying the suppressive functions of SKCM cells mediated by GSDMD remains elusive. Second, two BRAF^V600^ melanoma cell lines were used for this study, which may limit the generalizability of the results. Validation of other cell lines should be carried out in future studies.

## Conclusions

In this study, we constructed a prognostic model based on AIM2, CASP3, IL18, NLRP3, NOD2, GSDMA, GSDMC, GSDMD genes, which effectively predicted the prognosis of SKCM patients. Moreover, we screened different small molecular compounds according to the stratification of patients in the prognostic model. The results of in vitro experiments showed that overexpression of GSDMD and SB525334 treatment could suppress the proliferation, migration and invasion in SKCM cells.

### Supplementary Information

**Additional file 1: Figure S1.** GO/KEGG enrichment analysis of differntially expressed pyroptosis-related genes. Enriched GO terms in the BP (**A**), CC (**B**), MF (**C**) category, and KEGG pathway annotations (**D**). The right semicircle represents different functional categories, while the left semicircle consists of individual pyroptosis-related genes. **Figure S2.** Kaplan-Meier curves of the prognostic pyroptosis-related genes in the training cohort. The survival curves of AIM2 (**A**), CASP3 (**B**), GSDMA (**C**), GSDMC (**D**), GSDMD (**E**), IL18 (**F**), NLRP3 (**G**), and NOD2 (**H**). **Table S1.** List of 33 pyroptosis-related genes. **Table S2.** GO/KEGG functional enrichment of differntially expressed pyroptosis-related genes. **Table S3** 47 small molecular compounds were identified between low- and high-risk groups.

## Data Availability

All data and R script in this study are available from the corresponding author upon reasonable request. Publicly available datasets were analyzed in this study, these can be found in TCGA (https://portal.gdc.cancer.gov) and GTEx (https://www.gtexportal.org/home/) databases, and GEO (GSE65904).

## Abbreviations

SKCM: Skin cutaneous melanoma
TCGA: The cancer genome atlas
GTEx: Genotype-tissue expression
PRGs: Pyroptosis-related genes
TME: Tumor microenvironment
RNA-seq: RNA sequencing
PPI: Protein–protein interactions
GO: Gene ontology
BP: Biological process
CC: Cell composition
MF: Molecular function
KEGG: Kyoto encyclopedia of genes and genomes
OS: Overall survival
ICs: Immune checkpoints
IPS: Immunophenoscore
IHC: Immunohistology
IF: Immunofluorescence
