# Supplementary material for: System analysis based on the pyroptosis-related genes identifes GSDMD as a novel therapy target for skin cutaneous melanoma
Source: J Transl Med. 2023 Nov 10;21:801. doi: 10.1186/s12967-023-04513-9 (PMC10636830; doi:10.1186/s12967-023-04513-9)
Supplement: Supplementary file 1 — Additional file 1: Figure S1. GO/KEGG enrichment analysis of differntially expressed pyroptosis-related genes. Enriched GO terms in the BP (A), CC (B), MF (C) category, and KEGG pathway annotations (D). The right semicircle represents different functional categories, while the left semicircle consists of individual pyroptosis-related genes. Figure S2. Kaplan-Meier curves of the prognostic pyroptosis-related genes in the training cohort. The survival curves of AIM2 (A), CASP3 (B), GSDMA (C), GSDMC (D), GSDMD (E), IL18 (F), NLRP3 (G), and NOD2 (H). Table S1. List of 33 pyroptosis-related genes. Table S2. GO/KEGG functional enrichment of differntially expressed pyroptosis-related genes. Table S3 47 small molecular compounds were identified between low- and high-risk groups. [file 12967_2023_4513_MOESM1_ESM.docx]

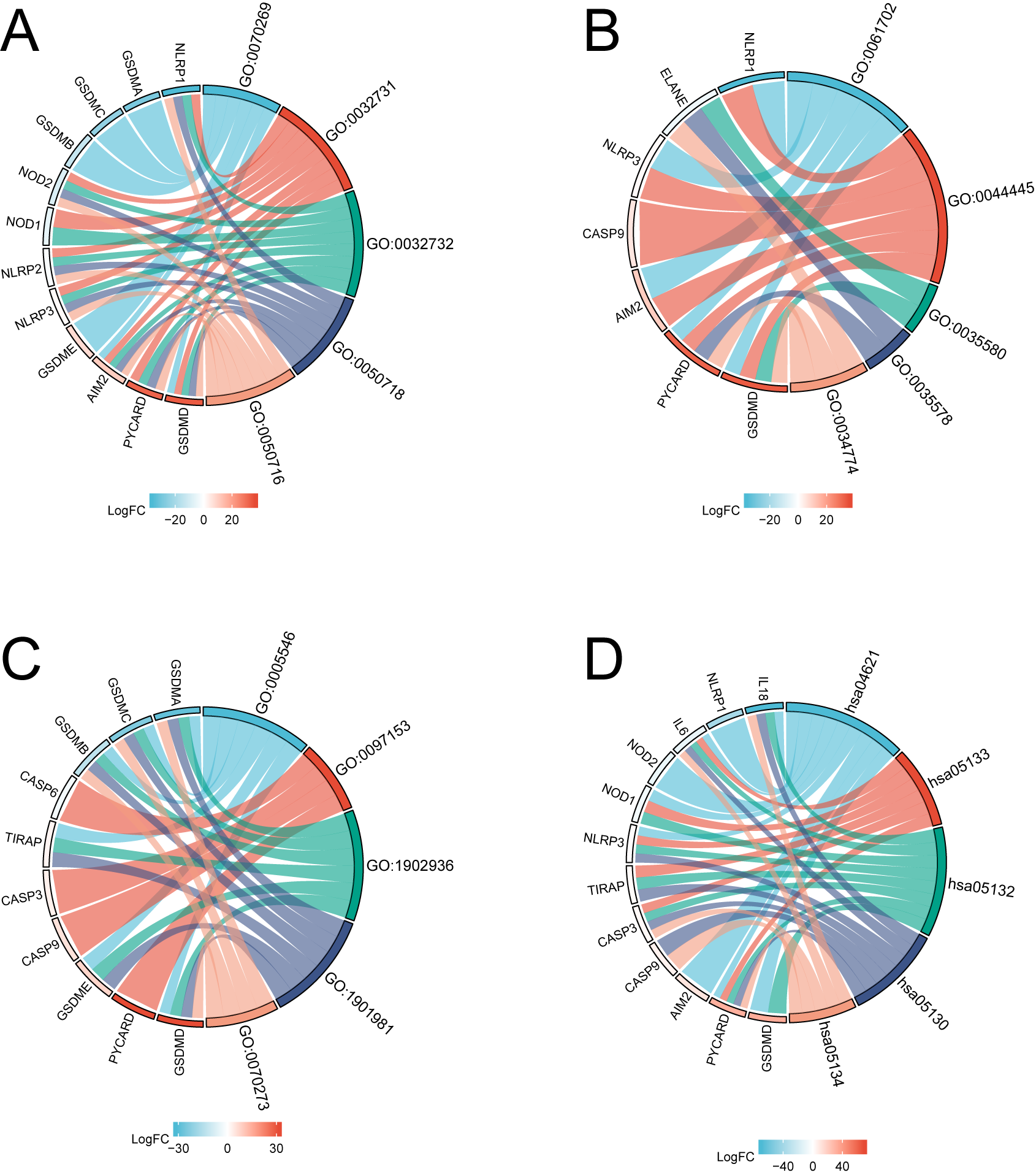


**Figure S1.** GO/KEGG enrichment analysis of differntially expressed pyroptosis-related genes. Enriched GO terms in the BP (A), CC (B), MF (C) category, and KEGG pathway annotations (D). The right semicircle represents different functional categories, while the left semicircle consists of individual pyroptosis-related genes.


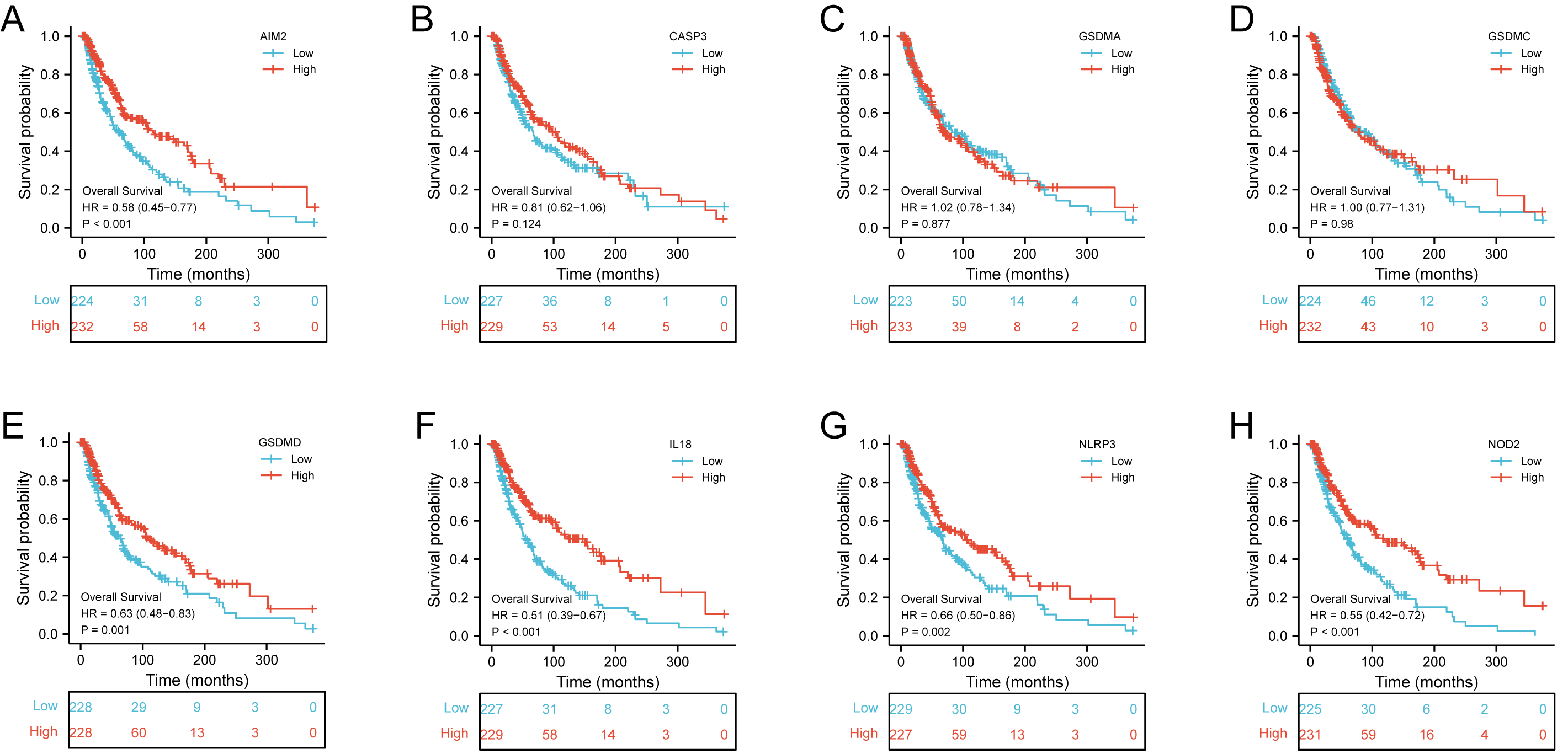


**Figure S2.** Kaplan-Meier curves of the prognostic pyroptosis-related genes in the

training cohort. The survival curves of AIM2 (A), CASP3 (B), GSDMA (C), GSDMC (D), GSDMD (E), IL18 (F), NLRP3 (G), and NOD2 (H).

**Table S1.** List of 33 pyroptosis-related genes

| **Genes** | **Full names** |
| --- | --- |
| AIM2 | Absent in melanoma 2 |
| CASP1 | cysteine-aspartic acid protease-1 |
| CASP3 | cysteine-aspartic acid protease-3 |
| CASP4 | cysteine-aspartic acid protease-4 |
| CASP5 | cysteine-aspartic acid protease-5 |
| CASP6 | cysteine-aspartic acid protease-6 |
| CASP8 | cysteine-aspartic acid protease-8 |
| CASP9 | cysteine-aspartic acid protease-9 |
| ELANE | elastase, neutrophil expressed |
| GPX4 | glutathione peroxidase 4 |
| GSDMA | gasdermin A |
| GSDMB | gasdermin B |
| GSDMC | gasdermin C |
| GSDMD | gasdermin D |
| GSDME | gasdermin E |
| IL18 | interleukin 18 |
| IL1B | interleukin 1 beta |
| IL6 | interleukin 6 |
| NLRC4 | NLR family CARD domain containing 4 |
| NLRP1 | NLR family pyrin domain containing 1 |
| NLRP2 | NLR family pyrin domain containing 2 |
| NLRP3 | NLR family pyrin domain containing 3 |
| NLRP6 | NLR family pyrin domain containing 6 |
| NLRP7 | NLR family pyrin domain containing 7 |
| NOD1 | nucleotide binding oligomerization domain containing 1 |
| NOD2 | nucleotide binding oligomerization domain containing 2 |
| PJVK | pejvakin/deafness, autosomal recessive 59 |
| PLCG1 | phospholipase C gamma 1 |
| PRKACA | protein kinase cAMP-activated catalytic subunit alpha |
| PYCARD | PYD and CARD domain containing |
| SCAF11 | SR-related CTD associated factor 11 |
| TIRAP | TIR domain containing adaptor protein |
| TNF | tumor necrosis factor |

**Table S2.** GO/KEGG functional enrichment of differntially expressed pyroptosis-related genes

| Ontology | ID | Description | Zscore | Ontology | ID | Description | zscore |
| --- | --- | --- | --- | --- | --- | --- | --- |
| BP | GO:0070269 | pyroptosis | 0.81649658 | CC | GO:0061702 | inflammasome complex | 1.34164079 |
| BP | GO:0032731 | positive regulation of interleukin-1 beta production | 0 | CC | GO:0044445 | cytosolic part | 1.63299316 |
| BP | GO:0032732 | positive regulation of interleukin-1 production | 0 | CC | GO:0035580 | specific granule lumen | 0 |
| BP | GO:0050718 | positive regulation of interleukin-1 beta secretion | 0.37796477 | CC | GO:0035578 | azurophil granule lumen | 0 |
| BP | GO:0050716 | positive regulation of interleukin-1 secretion | 0.37796477 | CC | GO:0034774 | secretory granule lumen | 0.57735027 |
| MF | GO:0005546 | phosphatidylinositol-4,5-bisphosphate binding | 0 | KEGG | hsa04621 | NOD-like receptor signaling pathway | -0.3333333 |
| MF | GO:0097153 | cysteine-type endopeptidase activity involved in apoptotic process | 1 | KEGG | hsa05133 | Pertussis | 0.81649658 |
| MF | GO:1902936 | phosphatidylinositol bisphosphate binding | 0 | KEGG | hsa05132 | Salmonella infection | 0.70710678 |
| MF | GO:1901981 | phosphatidylinositol phosphate binding | 0 | KEGG | hsa05130 | Pathogenic Escherichia coli infection | 1.13389342 |
| MF | GO:0070273 | phosphatidylinositol-4-phosphate binding | -1 | KEGG | hsa05134 | Legionellosis | 0.4472136 |

**Table S3** 47 small molecular compounds were identified between low- and high-risk groups.

| drug | FC | pValue |
| --- | --- | --- |
| AT13387 | 1.023601452 | 4.67E-23 |
| gemcitabine | 0.814500568 | 2.88E-19 |
| sirolimus | 0.781867211 | 1.10E-20 |
| teniposide | 0.747112445 | 9.67E-20 |
| clofarabine | 0.741084179 | 4.67E-18 |
| BRD-K01737880 | 0.708070027 | 9.74E-16 |
| topotecan | 0.699288108 | 9.77E-21 |
| navitoclax:birinapant (1:1 mol/mol) | 0.685346485 | 2.20E-19 |
| temsirolimus | 0.677374047 | 6.86E-27 |
| tretinoin:navitoclax (4:1 mol/mol) | 0.66951493 | 9.19E-19 |
| tanespimycin:gemcitabine (1:1 mol/mol) | 0.650059252 | 1.54E-12 |
| navitoclax:gemcitabine (1:1 mol/mol) | 0.649651445 | 2.36E-16 |
| ABT-737 | 0.6468775 | 3.71E-17 |
| etoposide | 0.621217288 | 2.67E-28 |
| ML162 | 0.57933818 | 3.82E-13 |
| alisertib:navitoclax (2:1 mol/mol) | 0.571932644 | 6.65E-11 |
| UNC0638:navitoclax (1:1 mol/mol) | 0.558713175 | 3.11E-17 |
| navitoclax:piperlongumine (1:1 mol/mol) | 0.555483423 | 3.62E-17 |
| mitomycin | 0.553968741 | 2.06E-21 |
| SNX-2112 | 0.551870502 | 3.95E-10 |
| vincristine | 0.529001096 | 8.73E-08 |
| decitabine:navitoclax (2:1 mol/mol) | 0.525902123 | 1.39E-10 |
| sirolimus:bortezomib (250:1 mol/mol) | 0.524561983 | 5.06E-14 |
| RITA | 0.512887121 | 7.15E-12 |
| SNX-2112:bortezomib (250:1 mol/mol) | 0.504230335 | 8.24E-14 |
| paclitaxel | 0.502887699 | 1.77E-07 |
| ML334 diastereomer | -0.101060701 | 1.91E-07 |
| BRD-K34099515 | -0.103497182 | 0.000431817 |
| JW-55 | -0.115141261 | 4.42E-11 |
| vandetanib | -0.11723373 | 0.000113923 |
| BRD-K97651142 | -0.117436292 | 0.0173201 |
| spautin-1 | -0.126211816 | 3.64E-11 |
| LY-2157299 | -0.128337592 | 2.56E-13 |
| FGIN-1-27 | -0.128687461 | 1.05E-13 |
| A-804598 | -0.131394073 | 7.09E-25 |
| austocystin D | -0.135231739 | 1.10E-19 |
| BRD-K02492147 | -0.138447478 | 6.67E-15 |
| ibrutinib | -0.139847877 | 0.008773019 |
| linsitinib | -0.144176819 | 2.20E-06 |
| BRD-K86535717 | -0.146548732 | 9.83E-18 |
| BRD-K09344309 | -0.148382598 | 3.32E-31 |
| BRD-K17060750 | -0.157505156 | 1.11E-30 |
| PD 153035 | -0.162979958 | 3.47E-06 |
| niclosamide | -0.183476662 | 2.88E-09 |
| VAF-347 | -0.186837927 | 2.21E-13 |
| SB-525334 | -0.191855967 | 7.21E-17 |
| SR8278 | -0.441523698 | 1.50E-13 |
